# Competency-based Training and the Competency Framework in Gynecology and Obstetrics in Brazil

**DOI:** 10.1055/s-0040-1708887

**Published:** 2020-05

**Authors:** Gustavo Salata Romão, Marcos Felipe Silva de Sá

**Affiliations:** 1 Department of Medicine, Universidade de Ribeirão Preto, Ribeirão Preto, SP, Brazil; 2 Department of Gynecology and Obstetrics, Faculdade de Medicina de Ribeirão Preto, Universidade de São Paulo, Ribeirão Preto, SP, Brazil

Although the idea of developing professional competency had been mentioned since the 1970s and 1980s,^1^ ^2^ the term *medical competency* remained undefined until the 2000s, when a broad literature review was published with the aim to clarify its meaning.^3^ In 2002, the result of this study was published in JAMA by Epstein and Hundert,^3^ and medical competency was defined as “the habitual and judicious use of communication, knowledge, technical skills, clinical reasoning, emotions, values, and reflection in daily practice for the benefit of the individual and the community to be served.” This made it clear how much the concept of competency differs from skill. The latter is used to designate the ability to perform specific technical-cognitive acts and actions such as intrauterine device insertion, obstetric examination, delivery care maneuvers, and the communication of bad news. The concept of competency is broader and includes the integration of knowledge, skills, and attitudes.^3^ ^4^ Therefore, medical competency is a multidimensional term and comprises a set of cognitive, technical, integrative, interpersonal, and affective-moral domains, as well as mental habits.^3^

Some aspects concerning the subcomponents of medical competency based on the definition of Epstein and Hundert (2002)^3^ stand out:

***Acquisition and use of knowledge:*** although evidence-based medicine is the major source of reliable knowledge for clarifying clinical doubts, the tacit, heuristic, and recognition-based knowledge is also greatly important for developing clinical competencies. Personal experience with clinical cases remains a great source of cognitive learning for physicians.^3^

***Integrative aspects of care:*** professional competency is not simply the demonstration of isolated clinical skills but their integration. When observing the medical activity as a whole, there is a difference between integrated technical skills and their isolated observation. A resident physician who evaluates the parturient according to the precepts of the partogram and demonstrates technical ability to perform delivery maneuvers at isolated times, may not provide adequate care during delivery when the integration of these two skills is needed. According to Friedman et al,^5^ a competent professional is able to think and act as a physician. Schon argues that professional competency is more than the ability to solve clinical problems using shortcuts and factual knowledge, but extends to the ability to deal with uncertain, challenging situations and make decisions from a limited set of information.^6^ Coping with these situations requires the mobilization of scientific, clinical, and humanistic expertise for guiding judgment and decisions.^7^ Stimulating reflection on the practice performed by the resident physician through feedback from his or her supervisor promotes the development of integrative skills for the integration of knowledge and proper management of uncertainties.^3^

***Interpersonal relationship and communication skills:*** the quality of the communication and relationship established between the physician and the patient interferes with health status, recovery, control of chronic diseases and treatment costs. Proper communication with patients favors a better understanding of their health conditions and a reduction in the degree of anxiety.^8^ The person-centered care is guided by qualified listening, response to their feelings, and the inclusion of patients and their values in the definition of the therapeutic plan. Teamwork skills are critical for patient safety and most medical errors result from failures in these skills.^3^

***Affective-moral dimensions:*** the evaluation of the affective-moral domain is a difficult one because it involves subjective characteristics, such as trust and professionalism. Neurobiological studies indicate the affective component is central for judgment and decision making; hence, emotional intelligence is a fundamental skill for medical practice.^3^

***Mental habits:*** professional competency requires mental habits that promote attention, investigative curiosity, self-awareness, and the initiative to recognize and correct one's own mistakes. Competent practitioners should be able to identify their degree of anxiety in the face of uncertainty and assess how much their emotional state may interfere with clinical judgment. From this point of view, medical errors can result from overestimated security in the face of uncertain situations.^3^

***Context:*** competency is context-dependent and involves the professional's personal skills, the patient's characteristics, the activity performed, the work environment and the health system in which the activity is inserted.^3^

The medical education reform movement has been a recurring theme in scientific literature and the subject of several proposals since the 1910 Flexner report.^9^ ^10^ ^11^ In the late 1990s, the ‘To err is human’ report published by the Institute of Medicine (IOM)^10^ in the United States showed that even with the 20^th^ century advances, healthcare in the country was not so safe. Such a report was produced from results of two large studies and showed estimates of preventable deaths caused by medical errors between 44,000 and 98,000 a year in the United States, that is, even the smallest estimates outnumbered deaths from traffic accidents, breast cancer, and AIDS.^10^

These results created a movement by society and regulatory agencies in the direction of placing competency-based medical education (CBME) as a priority.^1^ ^2^ ^9^ The aim of this change was to train better prepared professionals for the challenges of modern medicine. In the early 1990s, the Royal College of Physicians and Surgeons approved the CanMEDS Physician Competency Framework, which was revised and has been adopted as a standard for medical education in Canada since 2005.^12^ Following this trend, in 1999, the Accreditation Council for Graduate Medical Education (ACGME) developed the competency framework for undergraduate medical education in the United States (Outcome Project), which was adopted as a reference for medical education in that country in 2001.^13^ ^14^ The National Accreditation System of American medical schools was also restructured based on these competencies (the Next Accreditation System - NAS), and became effective in 2013.^15^ In the United Kingdom, the medical education reform had already begun in 1993, with publication of the ‘Tomorrow's doctor’ document. This document was revised in 2002,^16^ 2009,^17^ and 2018.^18^ The latter was called ‘Outcomes for graduates’, and corresponds to the current competency framework for undergraduate medicine in the UK.^18^ These countries were the first to require that residency programs were also competency driven.^2^ The certification exams for obtaining a specialist title and professional license have also become guided by competency assessment.^9^ ^13^ ^19^

This change meant the transition from a knowledge-based training system with exposure to specific content to a competency-based system. Training based on the acquisition of knowledge presupposes a predefined duration of the discipline so that a certain content can be assimilated by all learners. Competency-based training, on the other hand, establishes levels of competencies (milestones) that must be acquired and demonstrated by learners so they can progress independently from their peers. Knowledge-based training is more static and focused on disciplinary content, while competency-based training is more dynamic, learner-centered and requires greater flexibility in curricula and training programs. In knowledge-based training, the assessment process is performed at the end of each block, internship, or discipline with the aim to check content assimilation (“learning” assessment). In competency-based training, the assessment process is formative (“for learning” assessment), with the aim to check skills acquired from observing residents' performance, which includes, in addition to knowledge, the integration of procedural skills, communication and attitudinal components.^20^ The shift from knowledge-based to competency-based training has been considered the Flexnerian Revolution of the 21^st^ century.^9^

Worldwide, medical education and competency-based training are advancing for improving patient safety and training physicians committed to professionalism, continuing education, and social responsibility.^2^ ^11^ In 2014, the ACGME, the American Board of Obstetrics and Gynecology (ABOG), and the American Congress of Obstetricians and Gynecologists (ACOG), in partnership with the NAS, published the Milestones Project,^21^ a framework of core competencies to guide Gynecology and Obstetrics medical residency programs in the United States. The document has 28 competency axes hierarchized in 5 performance levels (milestones). Each milestone corresponds to a training stage that extends from apprentice level (level 1) to expert level (level 5).^21^ Other countries, such as Canada, the United Kingdom, Australia, and the Netherlands, have also published competency frameworks for gynecologist and obstetrician training based on essential competencies for professional practice in each country.^22^

In Brazil, the legislation also followed this trend. In 2016, the Federal Council of Medicine and the Ministry of Education established that **in all public notices of concourses for specialist titles in Brazil, the Brazilian Medical Association must observe the competency framework** and the minimum specialization training time.”^23^

Faced with the need to reorient and qualify the training of gynecologists and obstetricians in Brazil, the Scientific Board of the Brazilian Federation of Gynecologic and Obstetrics Associations (FEBRASGO) took the initiative to develop the first version of the Gynecology and Obstetrics Competency Framework in Brazil. In 2016, the initial version was developed from national references, such as curricular guidelines of the Gynecology and Obstetrics Medical Residency Programs,^24^ and international references, such as the ACOG Milestones Project.^21^ The initial document underwent a validation process involving an expert panel, in which more than 200 experts from 29 Febrasgo National Specialty Commissions carefully assessed the axes of competencies, issued opinions and suggestions through a structured form. In the light of experts' suggestions, the Gynecology and Obstetrics Competency Framework was completed with 21 Axes of Competencies, including Professionalism and Patient Safety. Competencies were hierarchized into three levels of complexity corresponding to the first, second, and third year of training in Gynecology and Obstetrics, with identification of their subcomponents as knowledge (K), skills (S), and attitudes (A).

In 2017, the Gynecology and Obstetrics Competency Framework was published in Portuguese^25^ and made available on the Febrasgo Web site. In 2018, this document was approved by the Brazilian Medical Association and the National Commission for Medical Residency and became the guide for Gynecology and Obstetrics Medical Residency Programs throughout Brazil.^26^

In 2019, the Brazilian Gynecology and Obstetrics Competency Framework was extensively revised and updated was updated by the FEBRASGO National Specialized Commissions, the FEBRASGO Medical Residency Commission and expert consultants.^27^

The updated version contains 16 Axes of Competence in Clinical and Surgical Obstetrics and Gynecology, including the Axes of Professionalism and Patient Safety.

Each axis presents the expected competencies for the resident at the end of the 1^st^ (R1), 2^nd^ (R2), and 3^rd^ (R3) years of residency in Gynecology and Obstetrics. The competencies for R2 are cumulative, compared with R1, and competencies for R3 are cumulative, with respect to R1 and R2. The subcomponents in each competency-axis were identified as Knowledge (K), Skills (S), or Attitudes (A) (Boxs 1 2 3 4 5 6 7 8 9 10 11 12 13 14 15 16).

**Box 1:** Healthcare and prenatal care

General goal: Acquisition of competencies related to low and high-risk obstetrical care and common complications during pregnancy.		
LEVEL	COMPETENCIES	COMPONENTS
**R1**	- Demonstrates knowledge of adaptations of women to pregnancy and changes in the pregnancy-puerperal cycle;	**K**
	- Demonstrates knowledge of the physiology of the fetus, placenta and annexes;	**K**
	- Demonstrates knowledge of national policies on maternal and child health and breastfeeding;	**K**
	- Demonstrates knowledge of the referral system for high-risk pregnancies;	**K**
	- Demonstrates basic knowledge and performs obstetrical care including clinical history, general physical, gynecological and obstetric examination with identification of risk factors;	**K / S / A**
	- Creates a bond and establishes effective communication with the pregnant woman and encourages the participation of the partner and/or family members of her choice in prenatal consultations;	**K / S / A**
	- Securely provides guidance to pregnant women and their families regarding common symptoms and signs in pregnancy, care with the diet, hygiene, clothing, physical and sexual activity, contraindicated medications, prenatal care, feeding, warning and labor signs, puerperal care, breastfeeding and postpartum contraception;	**K / S / A**
	- Understands and correctly completes the Pregnant Woman Card;	**K / S**
	- Correctly Interprets the screening tests results, including the assessment of fetal well-being;	**K / S**
	- Identifies and treats the main clinical complications during pregnancy;	**K / S / A**
	- Recognizes early the most prevalent clinical and obstetrical complications in pregnancy;	**K / S**
	- Initiates appropriate prophylaxis, including anti-D immunoglobulin in non-sensitized negative Rh pregnant women who present bleeding;	**K / S / A**
	- Appropriately handles equipment to assess fetal well-being (electronic fetal heart rate monitoring)	**S**
**R2**	- Appropriately manages the common prevalent medical and obstetrical complications in pregnancy such as anemia, urinary tract infection (UTI), gestational trophoblastic disease, hypertension, diabetes, infectious diseases, abnormal fetal growth and multiple pregnancy;	**K / S / A**
	- Appropriately manages patients with high-risk pregnancies based on current scientific evidence;	**K / S / A**
	- Manages or co-manages critically-ill pregnant women requiring care in an intensive care unit;	**S / A**
	- Recognizes uncommon medical and obstetrical complications and identifies indications for referral and/or transfer of care for these patients;	**K / S**
	- Performs tests for antepartum assessment of fetal well-being, such as electronic fetal heart rate monitoring and fetal biophysical profile;	**S**
	- Communicates effectively with patients and families regarding diagnostic and treatment invasive procedures in obstetrics, including amniocentesis, chorionic villus biopsy, cordocentesis and intrauterine fetal interventions;	**K / S**
	- Appropriately interprets the results of screening tests and invasive procedures (including prenatal genetic tests);	**K / S**
	- Identifies factors that hinder or contraindicate breastfeeding and guides appropriate care in these conditions;	**K / S**
	- Demonstrates knowledge of patient rights and responsibilities in cases of prenatally diagnosed anencephaly and other fetal abnormalities;	**K**
**R3**	- Demonstrates knowledge and recognizes atypical presentations of medical and obstetrical complications and their different treatment options;	**K**
	- Performs advanced fetal ultrasound for assessment of fetal morphology, Doppler flowmetry, placental location in patients with previous cesarean section to estimate the risk of placenta accrete, and invasive ultrasound-guided procedures such as amniocentesis;	**S**
	- Manages patients with bad obstetrical history, such as recurrent pregnancy loss and recurrent stillbirth; identifies the etiologies and proposes therapeutic measures;	**K / S**
	- Identifies atypical cases of gestational trophoblastic disease, formulates a differential diagnosis and proposes appropriate therapies;	**K / S**

Abbreviations: A, attitudes; K, knowledge; S, skills.

**Box 2:** Healthcare and intrapartum care

General goal: Acquisition of competencies related to low and high-risk obstetrical care and complications during labor and delivery.		
LEVEL	COMPETENCIES	COMPONENTS
**R1**	- Demonstrates up-to-date knowledge of obstetrical care based on evidence, safety and respect;	**K**
	- Demonstrates knowledge of pelvic floor anatomy, pelvic and perineum vascularization and innervation;	**K**
	- Demonstrates basic knowledge of the small pelvis anatomy and mechanism of labor and delivery;	**K**
	- Demonstrates knowledge of routine/ uncomplicated obstetrical care, including conduct of normal labor and delivery;	**K**
	- Demonstrates knowledge of appropriate indications for cesarean section;	**K**
	- Demonstrates knowledge of indications and contraindications to operative vaginal delivery (forceps and vacuum extractor);	**K**
	- Demonstrates knowledge of current public policies on childbirth care;	**K**
	- Identifies the signs and stages of labor, including preterm labor;	**S**
	- Demonstrates knowledge and classifies the pregnant woman according to Robson's criteria;	**K / S**
	- Provides appropriate intrapartum obstetrical care for women with uncomplicated pregnancies using the partograph and fetal well-being assessment methods;	**K / S / A**
	- Demonstrates knowledge and performs cervical ripening and labor induction;	**K / S**
	- Performs basic obstetrical skills in spontaneous vaginal delivery;	**K / S / A**
	- Differentiates between normal and abnormal labor;	**K / S**
	- Recognizes and preliminary manages intrapartum complications;	**K / S**
	- Identifies indications for transfer of care for patients with intrapartum complications;	**K / S**
	- Performs perineal protection and selective episiotomy;	**S**
	- Identifies and provides appropriate treatment for 1^st^ and 2^nd^ degree perineal lacerations;	**S**
	- Demonstrates ability to perform routine or low-complexity procedures for the immediate care for the newborn;	**K / S**
**R2**	- Demonstrates knowledge of risk factors for placenta accreta and the techniques maneuvers to be avoided in patients with such conditions;	**K**
	- Provides appropriate delivery support for patients with high-risk pregnancies;	**K / S / A**
	- Manages and provides appropriate delivery support for women with abnormal labor;	**K / S / A**
	- Manages intrapartum complications;	**K / S**
	- Performs operative vaginal delivery using forceps and vacuum extractor	**S**
	- Performs appropriate techniques maneuvers in breech presentation and shoulder dystocia;	**S**
	- Identifies and provides appropriate treatment for 3^rd^ and 4^th^ degree perineal lacerations;	**S**
**R3**	- Manages and provides appropriate care for women with intrapartum complex complications and conditions;	**K / S / A**
	- Provides intrapartum care for pregnant women with fetal abnormalities;	**K / S / A**
	- Performs rotational operative vaginal deliveries using forceps or vacuum extractor;	**S**

Abbreviations: A, attitudes; K, knowledge; S, skills.

**Box 3:** Healthcare and postpartum care

General goal: Acquisition of competencies related to low and high-risk postpartum care and its complications.		
LEVEL	COMPETENCIES	COMPONENTS
**R1**	- Demonstrates knowledge of normal postpartum care and common postpartum complications;	**K**
	- Demonstrates knowledge of risk factors, signs and symptoms of common postpartum complications;	**K**
	- Recognizes the need for closer monitoring of patients with delivery complications, such as bleeding and severe preeclampsia;	**K / S**
	- Demonstrates knowledge of the lactation physiology mechanisms and contraindicated drugs during breastfeeding;	**K**
	- Demonstrates knowledge of the main postpartum complications and implements preventive measures;	**K / S**
	- Requests and interprets histopathological analysis of abortion products;	**K / S**
	- Provides appropriate information and guidance on breastfeeding techniques, harmful practices and prevention of factors that hinder breastfeeding;	**K / S / A**
	- Manages complications related to breastfeeding (nipple trauma, breast engorgement, and puerperal acute mastitis);	**K / S / A**
	- Identifies postpartum mood disorders;	**K / S**
	- Properly identifies and manages common postpartum complications;	**K / S / A**
	- Provides appropriate care for the surgical wound and surgical site infection;	**K / S**
	- Performs lactation inhibition and induction based on formal indications;	**K / S**
	- Counsels patients about the risk of recurrence of prenatal, parturition and postpartum complications;	**K / S / A**
	- Performs insertion of intrauterine device (IUD) and subdermal implants intrapartum, after abortion and in the immediate postpartum period;	**S**
	- Provides appropriate guidelines for postpartum contraception;	**S / A**
**R2**	- Identifies and properly manages other factors that hinder breastfeeding (hypogalactia, cracked nipples, plugged ducts and breast abscess);	**K / S / A**
	- Identifies and provides initial care for postpartum mood disorders;	**K / S / A**
	- Identifies indications for interdisciplinary consultation, referral or transfer of care for patients with intrapartum complications;	**K / S**
	- Demonstrates knowledge and provides appropriate information and monitoring for patients with Gestational Trophoblastic Disease;	**K / S**
	- Manages or co-manages critically-ill women requiring care in an intensive care unit in the postpartum period;	**S / A**
**R3**	- Properly identifies and manages patients with complex complications of the postpartum period (such as severe hypertension, septic pelvic thrombophlebitis, and pulmonary embolism);	**K / S / A**

Abbreviations: A, attitudes; K, knowledge; S, skills.

**Box 4:** Technical skills in obstetric procedures

General goal: acquisition of competencies related to obstetrical procedures.		
LEVEL	COMPETENCIES	COMPONENTS
**R1**	- Performs pelvic examination for assessment of small pelvis, fetal presentations and cervical dilation;	**S**
	- Performs antepartum and intrapartum Electronic fetal monitoring;	**S**
	- Performs first trimester dilation, curettage, and suction in cases of incomplete spontaneous abortion;	**S**
	- Performs basic obstetrical skills in spontaneous vaginal delivery;	**S**
	- Performs perineal protection and selective episiotomy;	**S**
	- Performs repair of 1^st^ and 2^nd^ degree perineal or vaginal lacerations;	**S**
	- Performs primary cesarean section;	**S**
	- Performs insertion of intrauterine device (IUD) and subdermal implants intrapartum, after abortion and in the immediate postpartum period;	**S**
	- Applies trichloroacetic acid in genital warts;	**S**
**R2**	- Performs limited obstetrical sonography, gestational age assessment, and antenatal assessment (e.g., fetal presentation, fetal biometry, biophysical profile);	**S**
	- Performs uterine evacuation (medical and surgical) in cases of legal abortion or incomplete spontaneous abortion;	**S**
	- Performs second trimester uterine evacuation (medical and surgical) in incomplete spontaneous abortion;	**S**
	- Performs surgical treatment of ectopic pregnancy;	**S**
	- Performs external cephalic version for breech presentation;	**S**
	- Performs vaginal delivery in breech presentation and shoulder dystocia;	**S**
	- Performs operative vaginal delivery using forceps and vacuum extractor;	**S**
	- Performs appropriate management of 3^rd^ and 4^th^ degree perineal tears;	**S**
	- Performs repeat cesarean section;	**S**
	- Performs tubal ligation during cesarean delivery;	**S**
**R3**	- Performs cervical cerclage:	**S**
	- Performs detailed obstetrical sonography assessment (including Doppler, first and second trimester fetal anatomy, placental location following a previous cesarean section scar and prenatal diagnosis or therapeutic procedures such as amniocentesis);	**S**
	- Performs vaginal delivery in breech presentations (including the second twin);	**S**
	- Performs operative rotational vaginal delivery using forceps;	**S**
	- Identifies and manages uterine rupture or perforations;	**S**
	- Identifies and manages urinary bladder injuries;	**S**
	- Performs exploratory laparotomy and surgical treatment in cases of acute abdomen due to hemorrhage, including compressive sutures (B-Lynch and uterine artery ligation) and hysterectomy;	**S**
	- Performs cesarean sections in complex obstetrical complications (multiple repeat cesarean sections, placenta previa, placenta accreta, morbid obesity);	**S**

Abbreviation: S, skills.

**Box 5:** Technical skills in gynecological procedures

General goal: acquisition of competencies related to surgical techniques and gynecological perioperative management.		
LEVEL	COMPETENCIES	COMPONENTS
**R1**	- Demonstrates basic knowledge of abdominal, pelvic and perineal anatomy;	**K**
	- Demonstrates knowledge of basic surgical principles, including use of universal precautions and aseptic technique;	**K**
	- Demonstrates knowledge of laparoscopy, indications, advantages, limitations, and hemodynamic consequences;	**K**
	- Demonstrates knowledge of prophylactic strategies to reduce postoperative complications;	**K**
	- Demonstrates knowledge, performs preoperative care, and management of: medical comorbidities relevant to gynecologic surgery;	**K / S / A**
	- Positions patients appropriately for gynecological surgery;	**S**
	- Works effectively as a surgical assistant;	**S / A**
	- Demonstrates basic surgical skills including simple suturing and knots tying;	**S**
	- Performs simple abdominal incision and closure;	**S**
	- Performs simple vaginal or vulvar incision and repair, biopsy, Bartholin gland cyst or abscess excision or marsupialization);	**S**
	- Demonstrates knowledge, performs postoperative care, identifies and manages postoperative complications such as bleeding, infections, and thromboembolic events;	**K / S**
	- Performs intrauterine device and subdermal contraceptive implants insertion;	**S**
	- Performs wet mount microscopy for diagnosis of common vaginitis;	**K / S**
	- Performs simple biopsy of lesions of the lower female genital tract;	**S**
	- Performs treatment of external genital warts and vulvovaginal condylomatosis including application of trichloroacetic acid, podophyllin, imiquimod, and surgical excision;	**S**
	- Performs colposcopy-guided biopsy in cervical lesions;	**K / S**
**R2**	- Demonstrates knowledge of procedural indications for commons gynecologic co-morbidities;	**K**
	- Identifies and uses instruments and energy sources available for the procedure flow;	**K / S**
	- Demonstrates appropriate tissues handling;	**S**
	- Performs gynecological procedures by laparotomy (e.g., tubal ligation, unilateral or bilateral salpingo-oophorectomy, myomectomy, total and subtotal hysterectomy, Burch surgery);	**S**
	- Performs the initial management of surgical complications;	**S**
	- Performs vaginal surgery (e.g., repair of anterior and posterior vaginal wall prolapse, nymphoplasty, vaginal cysts excision);	**S**
	- Assembles and disassembles the basic set of video endoscopy (insufflation system, lighting and other equipment), verifies and adjusts the system parameters;	**S**
	- Works effectively as a surgical assistant in laparoscopy;	**S / A**
	- Performs simple laparoscopic incisions and suturing;	**S**
	- Performs umbilical puncture to create a pneumoperitoneum;	**S**
	- Performs proper insertion of laparoscopic instruments;	**S**
	- Properly uses laparoscopic optics of 0 and 30°;	**S**
	- Properly Inserts and handles cannulas and trocars;	**S**
	- Identifies risk situations and complications during surgery;	**S**
	- Performs the initial management of common postoperative complications such as bleeding, infections and thromboembolic events;	**S**
	- Performs treatment of low and high-grade cervical intraepithelial neoplasia (loop electrosurgical excision procedure [LEEP], cervical conization);	**S**
**R3**	- Performs complex gynecological procedures by laparotomy (e.g., total hysterectomy with adnexectomy);	**S**
	- Performs complex vaginal surgery (e.g., vaginal hysterectomy with or without prolapse, uterosacral ligament suspension, autologous and synthetic slings);	**S**
	- Performs basic endoscopic gynecological procedures (tubal ligation, adnexal surgeries and diagnostic hysteroscopy);	**S**
	- Works effectively as a surgical assistant in complex endoscopic gynecological procedures (e.g., adhesiolysis, salpingoplasty, myomectomy and operative hysteroscopy);	**S**
	- Demonstrates good intraoperative decision-making, including the ability to modify the initial surgical plan based on surgical findings;	**K / S / A**
	- Identifies and manages complex perioperative complications related to obstetrical and/or gynecological surgery, including the use of intraoperative interdisciplinary consultation;	**K / S / A**
	- Manages or co-manages critically-ill patients requiring care in an intensive care unit;	**K / S / A**
	- Performs surgical treatment of complex external genital warts and vulvovaginal condylomatosis (LEEP or extensive surgical exeresis);	**S**
	- Performs surgical treatment of benign and premalignant vulvar diseases;	**K / S / A**
	- Performs pelvic floor disorder investigations procedures, such as urodynamics and urethrocystoscopy;	**S**
	- Applies and adopts new scientific-based technologies;	**K / S / A**

Abbreviations: A, attitudes; K, knowledge; S, skills.

**Box 6:** Healthcare and care in pelvic floor disorders

General goal: acquisition of competencies related to care for women with pelvic floor disorders.		
LEVEL	COMPETENCIES	COMPONENTS
**R1**	- Demonstrates basic knowledge of anatomy and physiology of the pelvic floor;	**K**
	- Demonstrates knowledge of the pathophysiology of pelvic floor disorders, their signs, symptoms, and risk factors;	**K**
	- Formulates differential diagnosis of pelvic floor disorders;	**K / S**
**R2**	- Appropriately interprets the results of pelvic floor disorder investigations, such as ultrasound imaging in urogynecology, manometry, urodynamics, and urethrocystoscopy;	**K / S**
	- Performs the initial management of patients with uncomplicated pelvic floor disorders;	**K / S**
**R3**	- Properly performs investigation of pelvic floor disorders such as urodynamic study and urethrocystoscopy;	**S**
	- Performs the initial management of women with complex pelvic floor disorders;	**K / S**
	- Provides new care plans for patients with unsuccessful treatment for pelvic floor disorders;	**K / S**
	- Properly Identifies patients requiring multiprofessional treatment for pelvic floor disorders and leads the multiprofessional team;	**K / S / A**
	- Applies and adopts new evidence-based technologies;	**K / S / A**

Abbreviations: A, attitudes; K, knowledge; S, skills.

**Box 7:** Healthcare and gynecological oncology care

General goal: acquisition of competencies related to care for women with gynecological cancer.		
LEVEL	COMPETENCIES	COMPONENTS
**R1**	- Demonstrates knowledge of the etiology, prevention and risk factors of gynecological cancer;	**K**
	- Demonstrates knowledge of differential diagnostic methods of gynecological cancer (clinical, imaging and tumor markers);	**K**
	- Demonstrates basic knowledge of gynecological cancer treatment;	**K**
	- Provides appropriate guidance to patients on measures of primary prevention (including vaccination) and secondary prevention of cervical cancer;	**K / S / A**
	- Demonstrates knowledge and performs screening for cervical cancer, follow-up of patients with low-grade cervical cytological abnormalities, diagnosis (colposcopy and biopsy) and referral of patients with high-grade cervical intraepithelial neoplasia;	**K / S / A**
	- Indicates and performs endometrial, vaginal and vulvar biopsies on an outpatient care;	**K / S**
**R2**	- Demonstrates knowledge of hierarchical medical system for the management of patients with gynecological cancer;	**K / S / A**
	- Performs treatment of low and high-grade cervical intraepithelial neoplasia (loop electrosurgical excision procedure [LEEP], cervical conization);	**K / S**
	- Formulates differential diagnosis of benign, premalignant and malignant vulvar diseases;	**K / S**
	- Performs long-term follow-up of women treated for gynecological cancer;	**K / S**
	- Effectively communicates with patients with gynecological cancer and provides information about diagnosis, treatment options, and prognosis;	**K / S / A**
**R3**	- Demonstrates knowledge of comprehensive treatment plans for patients with gynecological cancer;	**K**
	- Demonstrates detailed knowledge of gynecological cancer regarding the different clinical presentations and specific therapeutic options;	**K**
	- Provides clinical and surgical treatment of benign and premalignant vulvar diseases;	**K / S / A**

Abbreviations: A, attitudes; K, knowledge; S, skills.

**Box 8:** Healthcare and care in contraception and family planning

General goal: acquisition of competencies related to family planning, indication, efficacy and safety of contraceptive methods.		
LEVEL	COMPETENCIES	COMPONENTS
**R1**	- Demonstrates basic knowledge of available contraceptive options;	**K**
	- Demonstrates knowledge of effectiveness, reversibility, risks, benefits, complications, contraindications and eligibility of the hormonal and non-hormonal contraceptive methods, including emergency contraception;	**K**
	- Demonstrates knowledge of the ethical and legal aspects of permanent contraceptive methods;	**K**
	- Provides basic counseling on the effectiveness, risks, benefits, complications and contraindications of available contraceptive methods and patients' preferences and conditions of use;	**K / S / A**
	- Properly prescribes and guides the use of reversible contraceptive methods;	**K / S / A**
	- Provides counseling on the effectiveness, risks, benefits, complications and contraindications of female and male sterilization;	**K / S / A**
	- Properly guides and provides referrals for couples desiring and/or meeting criteria for permanent contraceptive methods;	**K / S / A**
	- Performs intrauterine device (IUD) insertion procedure and subdermal contraceptive implantation;	**S**
**R2**	- Manages patients with medical diseases complicating the contraceptive methods use;	**K / S**
	- Performs tubal ligation;	**S**
**R3**	- Manages contraceptive complications and identifies patients who needs referral and/or transfer of care;	**K / S / A**

Abbreviations: A, attitudes; K, knowledge; S, skills.

**Box 9:** Healthcare and care in abnormal uterine bleeding

General goal: acquisition of competencies related to diagnosis and treatment in abnormal uterine bleeding.		
LEVEL	COMPETENCIES	COMPONENTS
**R1**	- Demonstrates basic knowledge of the mechanisms of normal and abnormal endometrial bleeding;	**K**
	- Demonstrates basic knowledge of the definition, structural and nonstructural causes of abnormal uterine bleeding;	**K**
	- Formulates differential diagnosis of abnormal uterine bleeding in different age groups;	**K / S**
	- Appropriately selects the tests and procedures necessary for the initial investigation of abnormal uterine bleeding;	**K / S**
	- Properly provides the initial treatment plan for the acute phase of abnormal uterine bleeding, including hemodynamic support;	**K / S**
	- Performs outpatient follow-up of patients with abnormal uterine bleeding;	**K / S / A**
	- Indicates and properly performs uterine curettage in cases of acute abnormal uterine bleeding;	**K / S**
**R2**	- Appropriately selects the exams and procedures necessary for the directed investigation of abnormal uterine bleeding (ultrasound, fractional curettage, magnetic resonance imaging, hormonal levels test and hemostasis evaluation);	**K / S**
	- Provides a treatment plan for each age group and clinical diagnosis;	**K / S / A**
	- Performs endometrial biopsy through different techniques, including fractional curettage;	**S**
	- Performs ultrasound to investigate abnormal uterine bleeding;	**S**
**R3**	- Demonstrates knowledge of new treatment options for abnormal uterine bleeding (including endometrial ablation and uterine artery embolization);	**K**
	- Provides new care plans for abnormal uterine bleeding refractory to initial treatment;	**K / S / A**
	- Performs procedures to investigate abnormal uterine bleeding such as diagnostic hysteroscopy and endometrial biopsy;	**S**
	- Works effectively as a surgical assistant in endoscopic gynecological procedures for abnormal uterine bleeding (myomectomy, polypectomy, endometrial ablation);	**S**
	- Performs hysterectomy for abnormal uterine bleeding refractory to other treatments;	**S**

Abbreviations: A, attitudes; K, knowledge; S, skills.

**Box 10:** Healthcare and care in endocrine gynecology

General goal: acquisition of competencies related to the diagnosis and treatment of endocrine-gynecological disorders and sexual dysfunctions.		
LEVEL	COMPETENCIES	COMPONENTS
**R1**	- Demonstrates basic knowledge of the physiology of the menstrual cycle, the relationships between the endocrine and reproductive systems and the sex steroid hormone metabolism;	**K**
	- Demonstrates basic knowledge of normal and abnormal pubertal development;	**K**
	- Demonstrates knowledge of bone metabolism, the hormonal and non-hormonal regulatory influences, the age-related disorders and the impact of the menopause transition on bone mass;	**K**
	- Demonstrates basic knowledge of female and male sexual response and biopsychosocial perspective of sexuality, including aspects of sexual orientation and gender identity;	**K**
	- Demonstrates basic knowledge of sexual dysfunctions;	**K**
	- Demonstrates basic knowledge of differential diagnoses of chronic pelvic pain;	**K**
	- Demonstrates basic knowledge of signs and symptoms related to common women's endocrine and metabolic disorders (amenorrhea, chronic anovulation and polycystic ovarian syndrome (PCOS), infertility, climacteric, premenstrual tension, dysmenorrhea, pelvic pain, endometriosis and obesity);	**K**
**R2**	- Formulates diagnosis and provides treatment of the main causes of chronic anovulation;	**K**
	- Demonstrates basic knowledge of the main drugs used in gynecological endocrinology, their indications and contraindications: estrogens, progestogens, androgens, GnRH agonists, SERMS (clomiphene, raloxifene, tamoxifen), letrozole, antiandrogens, bisphosphonates, denosumab, teriparatide, thyroxine, and bromocriptine;	**K**
	- Demonstrates basic knowledge of the definition of fecundity, fertility and infertility;	**K**
	- Demonstrates basic knowledge of the investigation of infertility and subfertility;	**K**
	- Formulates differential diagnosis, follow-up and multiprofessional therapeutic approach of women's endocrine and metabolic disorders, such as: disorders of puberty, amenorrhea, chronic anovulations and PCOS, Müllerian abnormalities, premature ovarian insufficiency, climacteric, infertility, osteoporosis, dysmenorrhea, chronic pelvic pain;	**K / S / A**
	- Formulates differential diagnosis, follow-up and multiprofessional therapeutic approach of female sexual dysfunction (dyspareunia, vaginismus, vulvodynia) and gender dysphoria;	**K / S / A**
**R3**	- Demonstrates basic theoretical and technical knowledge of methods to test hormonal levels (radioimmunoassay, enzyme immunoassay, chemiluminescence, electrochemiluminescence, high-performance liquid chromatography and mass spectrometry) and their inaccuracies (cross reaction, sensitivity, intra and inter-assay coefficients of variation). Demonstrates knowledge of stimulus tests for assessment of hypothalamic-pituitary-ovarian axis or investigation of adrenal gland disorders;	**K**
	- Demonstrates basic knowledge of the main therapeutic procedures used in Assisted Human Reproduction: intrauterine insemination (IUI), in vitro fertilization (IFV) and intracytoplasmic sperm injection (ICSI), including the general principles of techniques, ethical and legal issues, ovarian stimulation protocols and laboratory procedures;	**K**
	- Performs monitoring of patients in low-complexity assisted fertilization cycles;	**K / S**
	- Identifies indications for transfer of care for patients who need high complexity assisted reproductive treatment;	**K / S / A**
	- Formulates the differential diagnosis and therapeutic approach of patients with disorders of puberty;	**K / S**
	- Performs clinical management and follow-up of patients with vaginal agenesis and urogenital sinus malformations;	**K / S / A**

Abbreviations: A, attitudes; K, knowledge; S, skills.

**Box 11:** Healthcare and care in gynecologic and obstetric infections

General goal: acquisition of competencies related to diagnosis and treatment of infections in gynecology and obstetrics.		
LEVEL	COMPETENCIES	COMPONENTS
**R1**	- Demonstrates knowledge of the prevalent vertically transmitted infections: hepatitis B and C, herpes virus, HTLV I/II, influenza, rubella, toxoplasmosis, cytomegalovirus, HIV infection, syphilis, arboviruses;	**K**
	- Demonstrates knowledge and recommends appropriate vaccinations at each stage of the women's life;	**K / S**
	- Performs the initial assessment, formulates a differential diagnosis, and initiates treatment for the common infectious diseases in the obstetric and gynecologic outpatient care (low urinary tract infections (UTI), vulvovaginitis, genital ulcers, pelvic inflammatory disease (PID), puerperal and non-puerperal mastitis, latent syphilis and toxoplasmosis in pregnancy);	**K / S**
	- Performs and interprets the wet mount microscopy for diagnosis of common vaginitis;	**K / S**
	- Performs simple biopsy of lesions of the lower female genital tract;	**S**
	- Performs treatment of external genital warts and vulvovaginal condylomatosis including application of trichloroacetic acid, podophyllin, imiquimod and surgical excision;	**S**
	- Provides appropriate counseling on sexually transmitted infections (advise patient to bring their sex partners to medical evaluation, requests serology, respects confidentiality);	**K / S / A**
**R2**	- Demonstrates knowledge and recommends appropriate vaccinations in complex situations (HIV, autoimmune disease, immunosuppression, transplanted patient and women undergoing cancer treatment);	**K / S / A**
	- Performs the initial assessment, formulates a differential diagnosis, and initiates treatment for the infectious diseases in the obstetric and gynecologic hospital care (complicated mastitis, acute pelvic inflammatory disease (PID), tubo-ovarian abscess, infected abortion, endomyometritis, pyelonephritis, sepsis);	**K / S**
	- Performs gynecological, prenatal and postpartum follow-up of HIV-positive women;	**K / S / A**
**R3**	- Manages patients with complex infectious diseases (recurrent vulvovaginitis, infections refractory to initial treatment);	**K / S / A**
	- Performs surgical treatment of complex external genital warts and vulvovaginal condylomatosis (LEEP or extensive surgical exeresis);	**S**
	- Uses multidisciplinary approach for patients with complex infectious diseases;	**K / S / A**

Abbreviations: A, attitudes; K, knowledge; S, skills.

**Box 12:** Healthcare and care in gynecologic and obstetric emergencies

General goal: acquisition of competencies related to emergency care in gynecology and obstetrics.		
LEVEL	COMPETENCIES	COMPONENTS
**R1**	- Demonstrates basic knowledge of the most common gynecologic and obstetric emergencies;	**K**
	- Identifies, provides initial assessment (directed history and physical examination) and prehospital diagnosis of gynecological and obstetric emergencies;	**K / S / A**
	- Identifies and provides appropriate care for women with hypertensive emergencies and their complications in the intrapartum period;	**K / S / A**
	- Identifies and preliminary manages the obstetric emergencies such as umbilical cord prolapse, shoulder dystocia and acute fetal distress;	**K / S / A**
	- Identifies and preliminary manages the obstetric hemorrhage in the intrapartum period;	**K / S / A**
	- Demonstrates knowledge and preliminary manages the postpartum hemorrhage;	**K / S / A**
	- Provides prehospital management and identifies indications for consultation, referral, and/or transfer of care for women with gynecologic and obstetric emergencies;	**K / S / A**
**R2**	- Provides assessment, differential diagnosis and hospital-level care for women with gynecologic and obstetric emergencies;	**K / S / A**
	- identifies indications for surgical procedures in women with gynecologic and obstetric emergencies such as septic abortion, gestational trophoblastic disease, and ruptured ectopic pregnancy;	**K / S / A**
	- Performs uterine evacuation in cases of septic abortion, second trimester abortion or gestational trophoblastic disease;	**S**
	- Performs emergency cesarean section;	**S**
	- Performs operative vaginal deliveries using forceps in clinical and/or obstetric emergencies;	**S**
	- Appropriately manages women with septic or hemorrhagic shock;	**K / S / A**
	- Performs low-complexity invasive procedures in postpartum hemorrhage such as uterine balloon placement;	**S**
	- identifies indications for puerperal hysterectomy and the use of blood components and blood-derived products in postpartum hemorrhage;	**K / A**
	- Demonstrates knowledge and provides appropriate care to victims of sexual violence;	**K / S / A**
**R3**	- Provides hospital-level assessment, formulates differential diagnosis and performs surgical treatment in complex emergencies such as placental detachment, placenta accreta, and uterine rupture;	**K / S / A**
	- Appropriately manages women with treatment-refractory postpartum hemorrhage and properly indicates blood transfusion and/or damage control surgery;	**K / S / A**
	- Manages or co-manages critically ill patients, provides vasoactive drugs management, volume replacement and performs life support maneuvers such as cardiorespiratory resuscitation, and orotracheal intubation;	**K / S**
	- Performs exploratory laparotomy and surgical treatment in cases of acute abdomen due to hemorrhage, including compressive sutures (B-Lynch and uterine artery ligation) and hysterectomy;	**S**
	- Provides appropriate information and monitoring for patients with gestational trophoblastic disease, including locally advanced or metastatic disease;	**K / S**

Abbreviations: A, attitudes; K, knowledge; S, skills.

**Box 13:** Healthcare and care in breast disorders

General goal: acquisition of competencies related to the care of women with breast disorders.		
LEVEL	COMPETENCIES	COMPONENTS
**R1**	- Demonstrates knowledge of the most common benign and malignant breast disorders (cyclic and noncyclic breast pain, fibrocystic breast changes, cysts and lumps, nipple discharge and breast cancer);	**K**
	- Performs assessment, formulates differential diagnosis and provides initial treatment in common benign breast disorders (cyclic and noncyclic breast pain, fibrocystic breast changes, cysts and lumps, nipple discharge and breast cancer);	**K / S**
	- Identifies women with breast cancer risk factors in primary care settings by using clinical information and breast cancer (BC) risk assessment models, and provides a care plan for follow-up these patients;	**K / S**
	- Guides the breast cancer screening in primary care settings and properly interprets imaging results using the BI-RADS classification;	**K / S**
	- Performs low-complexity procedures (puncture of cysts or abscesses, including ultrasound-guided puncture);	**K / S**
**R2**	- Demonstrates knowledge of the breast cancer treatment options and the treatment sequence pacing;	**K**
	- Performs initial investigation procedures of breast disorders (puncture/aspiration biopsy of cysts and breast lumps, percutaneous core-needle breast biopsy under direct visualization);	**S**
	- Performs low-complexity surgical procedures in the treatment of benign mammary disorders (duct excision, palpable lump excision);	**S**
	- Provides care plans, follow-up and referral for patients with abnormalities in clinical examination, mammography or ultrasound, and when necessary, indicates the appropriate type of biopsy;	**K / S / A**
**R3**	- Purposes primary prevention measures for high-risk breast cancer patients;	**K / S**
	- Uses multidisciplinary and hierarchical approach (at primary, secondary and tertiary levels of care) for patients with complex breast diseases;	**K / S / A**
	- Performs high-complexity surgical procedures in the treatment of breast diseases, such as segmental resection, total mastectomy (simple) and accessory axillary breast excision, non-palpable lesions biopsy, fistulectomy and surgical approach to the treatment for gynecomastia;	**S**
	- Provides appropriate assistance and follow-up in the postoperative period of breast surgeries (cancer or not);	**K / S**
	- Purposes follow-up measures for patients undergoing breast cancer treatment;	**K / S**

Abbreviations: A, attitudes; K, knowledge; S, skills.

**Box 14:** Healthcare and care in non-reproductive disorders

General goal: acquisition of competencies related to the care of non-reproductive disorders		
LEVEL	COMPETENCIES	COMPONENTS
**R1**	- Demonstrates knowledge of the common non-reproductive medical disorders related to the reproductive system (hypertension, diabetes, dyslipidemia, metabolic syndrome, obesity, anorexia, depression, osteoporosis, lupus, thyroid dysfunction, HIV infection);	**K**
	- Performs anamnesis, physical examination, differential diagnosis and appropriately request the exams and procedures for the initial diagnostic approach;	**K / S**
**R2**	- Interprets test results for the disorders described above;	**K / S**
	- Preliminary manages the therapeutic plans for patients with common non-reproductive medical disorders (hypertension, diabetes, thyroid dysfunction);	**K / S / A**
**R3**	- Preliminary manages the therapeutic plans for patients with complex non-reproductive medical disorders (osteoporosis, metabolic syndrome, eating disorders, HIV infection);	**K / S / A**

Abbreviations: A, attitudes; K, knowledge; S, skills.

**Box 15:** Patient safety in gynecology and obstetrics

General goal: acquisition of competencies related to patient safety and assimilation of this culture among health professionals and services in the country.		
LEVEL	COMPETENCIES	COMPONENTS
**R1**	- Appropriately utilizes the national protocols for checking and promoting patient safety: patient identification, effective team communication in the professional setting, medication safety, surgical safety checklist, hand hygiene practice, reduction of risk for falls and pressure ulcers;	**K / S / A**
	- Demonstrates knowledge of the medical errors' epidemiology and the differences between near misses, near accident, accident, adverse event, sentinel event and medical error;	**K**
	- Demonstrates knowledge of the work routine and care protocols of the institution;	**K**
	- Recognizes the importance of medical record quality as a determinant in patient safety. Records all the pertinent information to the case;	**K / S / A**
	- Obtains the informed consent form to perform procedures;	**K / S / A**
**R2**	- Performs the transfer of care to other professionals and to the various points of care appropriately (transitions of care);	**K / S / A**
**R3**	- Participates in patient safety reports and analysis of surveillance systems;	**S / A**
	- Reports errors and near misses to the institutional surveillance system and superiors in charge;	**S / A**
	- Actively participates in improving the quality of patient safety in the workplace environment;	**S / A**

Abbreviations: A, attitudes; K, knowledge; S, skills.

**Box 16:** Professionalism in gynecology and obstetrics

General goal: acquisition of competencies related to professionalism in Gynecology and Obstetrics.		
LEVEL	COMPETENCIES	COMPONENTS
**R1**	- Shows respect and interest in patients as human beings, regardless of their sexual choice, sex, religion, race/color, and social class;	**A**
	- Communicates effectively with patients, family members, caregivers and work team members and does not use derogatory or aggressive language;	**S / A**
	- Promotes qualified listening without prejudice and discusses the care plan with the patient and the work team;	**S / A**
	- Meets the patients' needs by accepting their inconveniences;	**A**
	- Maintains appropriate limits on relationships with patients, family members and colleagues;	**A**
	- Demonstrates punctuality and proper time management;	**A**
	- Demonstrates awareness of one's own limitations and improvement needs;	**A**
	- Requests and accepts feedback from supervisors and preceptors;	**A**
	- Accepts feedback from peers and patients, and respects differences of opinion;	**A**
	- Maintains composure even in difficult situations;	**A**
	- Maintains appropriate appearance in accordance with institutional standards and national resolutions;	**A**
	- Ensures medical confidentiality in all situations in which patients are involved, does not convey their information, photos or imaging, including social media, according to the current legislation;	**A**
	- Knows and respects the current legislation regarding the use of image in scientific publications;	**K / A**
	- Respects rules and regulations of the health system;	**K / A**
	- Has a hierarchical commitment to other residents and preceptors;	**A**
	- Demonstrates understanding and correctly interprets the Medical Ethics Code, the resolutions of the medical councils, and the national legislation;	**A**
	- Is cooperative with the work team;	**A**
**R2**	- Works effectively in interprofessional and interdisciplinary health teams;	**A**
	- Considers scientific evidence in decision making;	**K / A**
	- Collaborates, assists and supports junior residents in their learning process and technical skills development;	**A**
	- Effectively supervises and educates lower-level residents	**A**
**R3**	- Incorporates risk management into the communication process and role models effective communication to junior colleagues;	**A**
	- Communicates effectively with patients and family members in more complex situations (bad news);	**K / S / A**
	- Demonstrates leadership and conflict mediation skills;	**S / A**
	- Effectively supervises and educates lower-level residents;	**A**

Abbreviations: A, attitudes; K, knowledge; S, skills.

## Conclusion

Brazil is a country of continental dimensions that presents great regional differences in terms of the availability of human and economic resources as well as in the social profile of patients. The orientation of residency programs by the Competence Framework will demand a lot of attention from supervisors and preceptors, not only in the transfer of technical knowledge, but also in the reorientation of practices and in communication with other professionals. It is necessary for the educational institutions that maintain the residency programs to make investments in infrastructure and the training of preceptors and supervisors. Competency-based medical education is an integrated, progressive, learner-centered training strategy focused on assessment of the expected performance for the physician's clinical practice. It emphasizes learners' continuing education and accountability for their development through formative assessment. The Gynecology and Obstetrics Competency Framework is an important reference for harmonizing and qualifying medical residency programs throughout Brazil, as well as guiding the resident's training and evaluation processes, and the certification of Gynecology and Obstetrics specialists.
